# Polo-like Kinase 1 Expression as a Biomarker in Colorectal Cancer: A Retrospective Two-Center Study

**DOI:** 10.3390/biomedicines13010054

**Published:** 2024-12-29

**Authors:** Lana Jajac Brucic, Vesna Bisof, Majana Soce, Marko Skelin, Ivan Krecak, Andjela Nadinic, Branka Vrbicic, Zivana Puljiz, Suzana Hancic, Slavko Gasparov

**Affiliations:** 1 Department of Hematology, Oncology, Allergology and Clinical Immunology, General Hospital of Sibenik-Knin County, 22000 Sibenik, Croatia123nadinic@gmail.com (A.N.); 2 Department of Oncology, University Hospital Center Zagreb, 10000 Zagreb, Croatia; 3 School of Medicine, University of Zagreb, 10000 Zagreb, Croatia; 4 Pharmacy Department, General Hospital of Sibenik-Knin County, 22000 Sibenik, Croatia; 5 Department of Pathology and Cytology, General Hospital of Sibenik-Knin County, 22000 Sibenik, Croatia; 6 Department of Biochemical Engineering, Faculty of Food Technology and Biotechnology Zagreb, 10000 Zagreb, Croatia; 7 Institute of Clinical Pathology and Cytology, Merkur University Hospital, 10000 Zagreb, Croatia; 8 Department of Pathology, Medical School Zagreb, University of Zagreb, 10000 Zagreb, Croatia

**Keywords:** colorectal cancer, polo-like kinase 1, metastasis, targeted therapy

## Abstract

**Background/Objectives**: Early-onset colorectal cancer (EOCRC) is more frequently characterized by poorly differentiated, aggressive tumors, often diagnosed at advanced stages, and associated with worse prognoses. Despite these differences, current treatment guidelines do not distinguish between EOCRC and late-onset colorectal cancer (LOCRC). Elevated expression of polo-like kinase 1 (PLK-1) has been linked to advanced disease stages and poorer treatment outcomes, including resistance to both chemotherapy and radiotherapy. However, data on PLK-1 expression in EOCRC compared to LOCRC remain limited. **Methods**: Patients with sporadic CRC, aged >18 years, were included in this study. We categorized the patients into two groups: patients younger than 50 years, and those aged 50 years or older. Immunohistochemical staining was performed to assess PLK-1 expression. The aim of this study was to assess PLK-1 expression considering the age of the patients and its effects on overall survival (OS) and progression-free survival (PFS). **Results**: A total of 146 patients with metastatic colorectal cancer (mCRC) were included in this retrospective two-center study. Patients with low PLK-1 expression were older than patients with high PLK-1 expression (64 (49–71) years vs. 49 (42–67) years, *p* = 0.016). Multiple logistic regression confirmed that age is a significant predictor of PLK-1 expression, independent of the covariates (*p* = 0.036). The Kaplan–Meier analysis revealed no significant association between PLK-1 expression and PFS (*p* = 0.397) or OS (*p* = 0.448). Accordingly, Cox proportional hazards regression analysis showed no significant association between PLK-1 expression and OS (HR 1.20, 95% CI 0.73–1.96, *p* = 0.598) or PFS (HR 0.85, 95% CI 0.51–1.43, *p* = 0.611) when covariates were taken into account. Finally, no significant differences in PFS (*p* = 0.423) or OS (*p* = 0.104) were found between the age groups of interest. **Conclusions**: PLK-1 expression was not associated with survival or progression in EOCRC and LOCRC patients. Further research on these combinations is necessary, as well as the discovery of new potential targets for targeted therapy and the mechanisms of synergistic effects in tumors with PLK-1 overexpression.

## 1. Introduction

Colorectal cancer (CRC) is the third-most-common cancer worldwide and the second-leading cause of cancer-related deaths. In Europe, it ranks second in both incidence (following breast cancer) and mortality (following lung cancer). The highest incidence rates are observed in developed countries; however, there is a notable trend of increasing incidence in low- and middle-income countries, indicative of the globalization of lifestyle-related risk factors such as unhealthy diets, physical inactivity, and obesity [1,2].

The risk of developing colorectal cancer (CRC) increases with age. Approximately 90% of cases occur after the age of 50, referred to as late-onset colorectal cancer (LOCRC), with the risk tripling after the age of 65. The median age for the onset of colon cancer is 68 years for men and 72 years for women, whereas the median age for rectal cancer patients is 63 years for both genders [1,3,4]. Most researchers set the age threshold for early-onset colorectal cancer (EOCRC) at 50 years; however, this boundary is not clearly defined, with some considering lower thresholds such as 45 or 40 years. This important patient cohort remains poorly understood because, in the majority of studies, these patients are not analyzed as a separate group but, rather, treated and evaluated alongside the general population. Approximately 30% of EOCRC cases carry hereditary mutations linked to cancer predisposition syndromes (e.g., hereditary nonpolyposis colorectal cancer), 20% have familial CRC, and the remaining 50% are sporadic cases. In recent decades, both the overall incidence and mortality of CRC in Europe and the United States have declined, particularly among those aged over 50 years, largely attributed to the implementation of early detection screening programs [2,4,5]. Conversely, despite the early appearance of symptoms in EOCRC, patients are often referred for further evaluation late, resulting in diagnoses at more advanced stages. On average, there is a delay of 4 to 6 months in diagnosing EOCRC, with some case reports describing delays of up to 2 years. This delay is, on average, 1.4 times longer compared to older patients [4,6,7,8,9,10,11].

Although the data are inconsistent, EOCRC appears to have a worse prognosis compared to older counterparts, characterized by more frequent local recurrences and distant metastases [12,13,14,15,16,17,18,19]. The unfavorable behavior of CRC in the younger population necessitates the exploration of tailored therapies that may improve outcomes. Consequently, several studies investigating new prognostic and predictive factors are currently underway to identify better therapeutic options. One such factor is polo-like kinase 1 (PLK-1), a mitotic serine/threonine kinase and cell-cycle regulator that is essential for cell division. PLK-1 plays a crucial role in regulating cell entry into mitosis, spindle formation, and cytokinesis [20,21]. Although the functional significance of PLK-1 in carcinogenesis and malignant progression is not yet fully understood, its inhibition has been shown to result in growth cessation or apoptosis of cancer cells in previous reports [22,23]. Furthermore, overexpression of PLK-1 has been identified in various types of cancer, including CRC, and is associated with tumor size, depth of invasion, lymph node involvement, and poorer survival outcomes [21,22,24].

Thus, in the present study, we aimed to compare PLK-1 expression among different groups of patients with mCRC, with a particular emphasis on the differences in expression between EOCRC and LOCRC. Additionally, we sought to establish whether PLK-1 expression and age can predict outcomes in these patients independent of potential confounders.

## 2. Materials and Methods

### 2.1. Study Design and Participants

In this retrospective two-center study, we included 146 patients with mCRC treated at the General Hospital of Sibenik-Knin County (85 patients) and/or the University Hospital Zagreb (61 patients) from 1 January 2015 to 31 December 2020. Patients with sporadic CRC, aged >18 years, were included in the present study. The following patients were excluded from this analysis: patients with hereditary polyposis CRC and syndromic CRC. The patients were divided into two groups: younger patients with EOCRC (diagnosed at <50 years), and older patients with LOCRC (diagnosed at ≥50 years). For each included patient, clinical and pathological features were collected through a review of medical records. The primary objective was to analyze PLK-1 protein expression in younger and older mCRC patients. The secondary objectives were to determine the association between PLK-1 expression and OS/PFS and to examine the differences in PLK-1 expression and clinical and pathological characteristics in younger and older mCRC patients.

This study was conducted in accordance with the Declaration of Helsinki and was approved by the ethics committees of the participating institutions.

### 2.2. Tissue Sampling and Preparation

**Figure 1:**
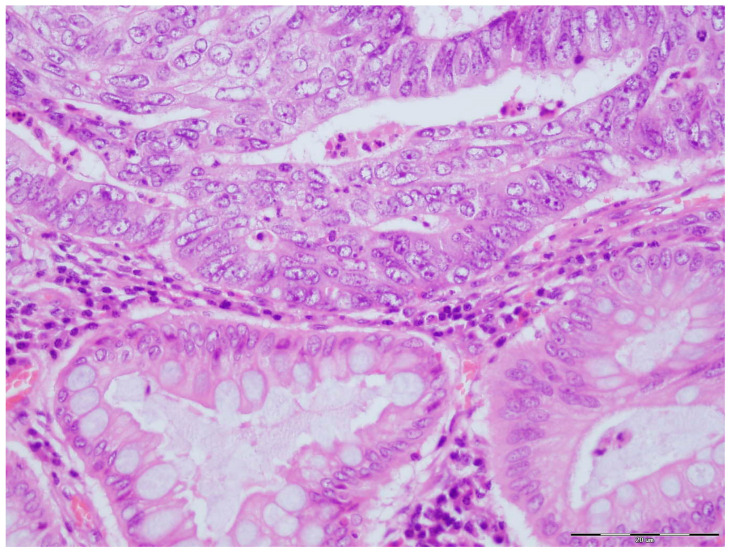
Hematoxylin and eosin staining of colorectal cancer.

For this study, archival tissue samples from primary tumors (formalin-fixed, paraffin-embedded) were used. The diagnosis of colorectal cancer was reviewed on histological slides stained with hematoxylin and eosin (H&E). Paraffin-embedded tissue blocks were sectioned at a thickness of 3 μm using a rotary microtome (RM2065 SuperCut, Leica Biosystems, Nussloch, Germany ) and stained via the standard H&E method in an automated Tissue-Tek DRS 2000 system (Sakura Finetek Europe, Alphen aan den Rijn, The Netherlands) (Figure 1).

### 2.3. PLK-1 Immunohistochemistry

Standard immunohistochemical staining was performed on tumor tissue samples to determine PLK-1 protein expression using a specific antibody. Three-micron-thick tissue sections were processed with a buffer solution (pH 9.0) at 97 °C for 20 min, during which deparaffinization, rehydration, and epitope retrieval were completed. This entire process was conducted under high pressure in a PT Link device (Dako/Agilent, Santa Clara, CA, USA), using the Heat-Induced Epitope Retrieval (HIER) method. After cooling and rinsing with neutral buffer solutions, primary antibody incubation was carried out. The procedure continued in an automated immunohistochemical stainer (Autostainer LINK 48, Dako/Agilent, Santa Clara, CA, USA). For PLK-1 expression assessment, a monoclonal rabbit anti-PLK-1 primary antibody (ab155095, Abcam, Waltham, MA, USA) was used at a 1:500 dilution, with a 45 min incubation. Visualization of the antigen–antibody complex was achieved using a secondary antibody (EnVision, Dako/Agilent, Santa Clara, CA USA), followed by 3,3′-diaminobenzidine (DAB) chromogen. Finally, the samples were counterstained with hematoxylin.

**Figure 2:**
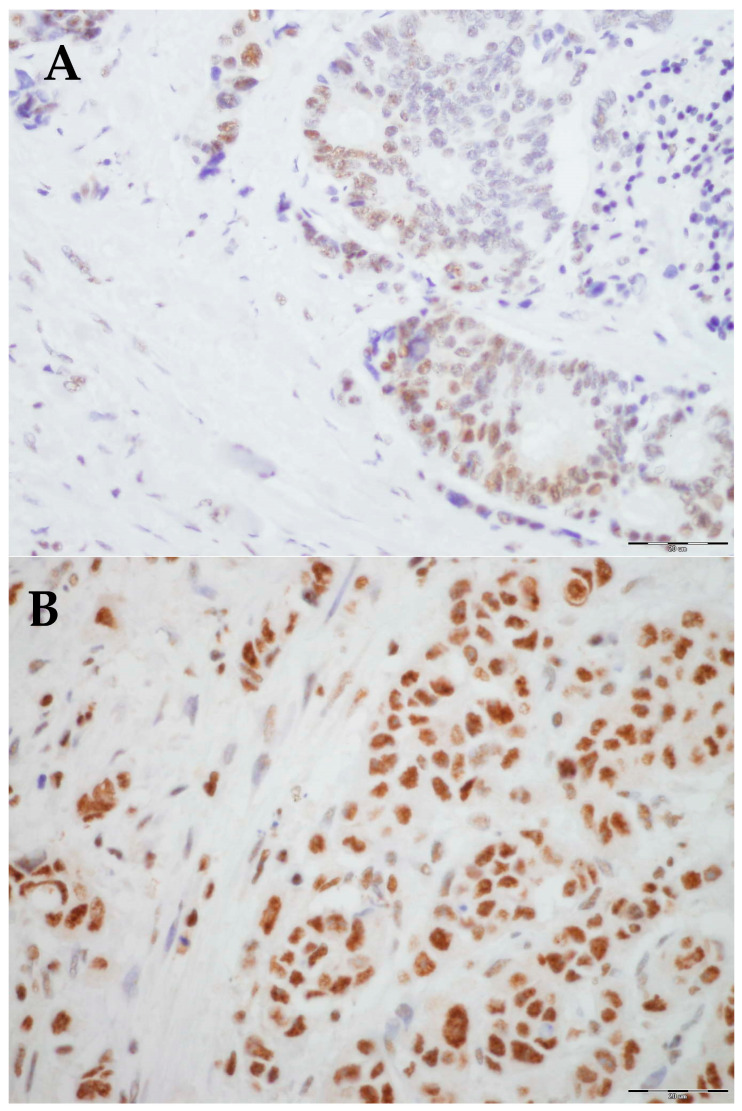
Representative histological images of low and high expression of PLK-1: (**A**) Low expression, 40× magnification. (**B**) High expression, 40× magnification.

Immunohistochemical staining results were analyzed using an Olympus BX51 microscope at 40× and 400× magnifications across five fields of view (Figure 2). The results were interpreted using a weighted score, calculated by multiplying the percentage of positive tumor cells by the staining intensity, yielding a score ranging from 0 to 12 (Table 1) [25].

The immunohistochemical staining results were evaluated by assessing both nuclear and cytoplasmic positivity in tumor cells. The percentage of reactive cells was graded on a scale from 0 to 4: 0 = 0–5%, 1 = 6–25%, 2 = 26–50%, 3 = 51–75%, and 4 = more than 75% of cells. The staining intensity was rated on a scale from 0 to 3: 0 = no reaction, 1 = weak reaction (1–50% of cells visible at 400× magnification), 2 = moderate intensity (1–50% of cells visible at 40× magnification or >50% of cells visible at 400× magnification), and 3 = strong intensity (51–100% of cells visible at 40× magnification).

### 2.4. Statistical Analysis

For data analysis and graphical data representation, Prism 10 for Windows^®^ (version 10.3.1, GraphPad, La Jolla, CA, USA) and JASP for Windows^®^ (version 0.19.0, Amsterdam, The Netherlands) were used. Quantitative variables were expressed as the mean ± standard deviation or median (interquartile range), depending on the distribution of the data. Independent quantitative variables were compared using either Student’s *t*-test or the Mann–Whitney U test, as appropriate. To assess whether age was a significant predictor of PLK-1 expression independent of covariates such as sex, tumor grade, or treatment with biological therapy, multiple logistic regression analysis was performed. Kaplan–Meier analysis was employed to examine whether PLK-1 intensity and patient age were associated with progression-free survival (PFS) and overall survival (OS) in CRC patients. Lastly, to determine whether PLK-1 was independently associated with these outcomes, controlling for age, sex, ECOG status, and the presence of metastasis at diagnosis, a Cox proportional hazards regression was utilized. Statistical significance was set at *p* < 0.05 for all analyses.

## 3. Results

A total of 146 patients with metastatic colon cancer were included in this analysis; 50% of the patients were female, and their average age at the time of diagnosis was 56 years. Patients were followed up for a median of 37 months. Relevant baseline characteristics between the two groups of interest are delineated in Table 2. The older subgroup of patients had a higher percentage of male patients, worse ECOG status, and more frequently had the caecum, ascending colon, and sigmoid colon as primary tumor locations. In contrast, younger patients more commonly had high-grade tumors, peritoneal metastasis, and the rectum as the primary tumor location compared to their older counterparts.

**Figure 3:**
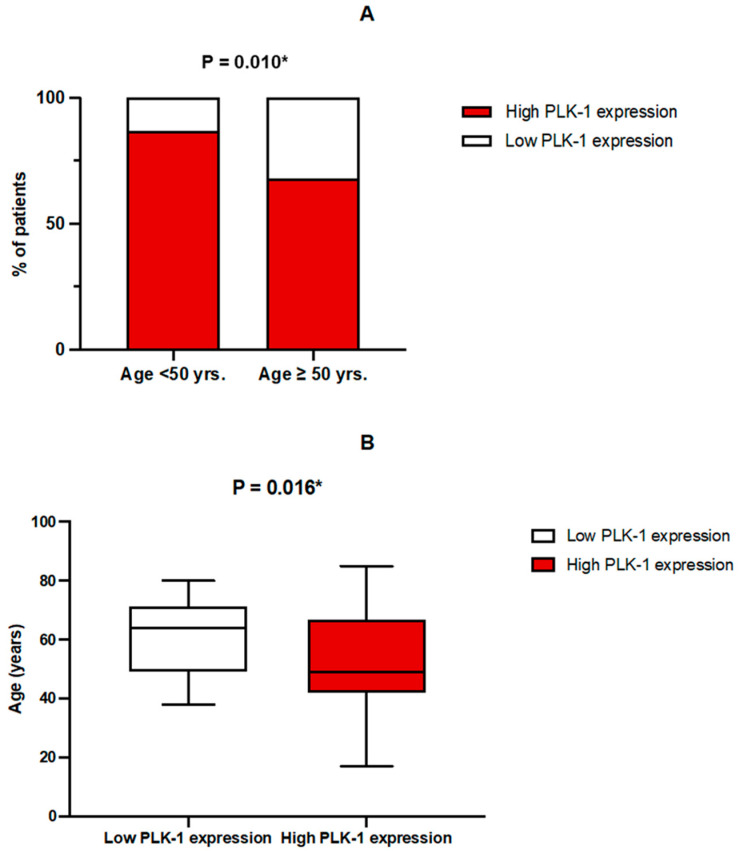
(**A**) Comparison of PLK-1 expression between age groups; * chi-squared test. (**B**) Comparison of age depending on PLK-1 expression; * Mann–Whitney U test.

High PLK-1 expression was more prevalent in the younger subgroup of patients (<50 years) (58 (86.6%) vs. 54 (68.4%), *p* = 0.010) (Figure 3A). Patients with PLK-1 expression deemed as low were older than patients in the subgroup with high PLK-1 expression (64 (49–71) years vs. 49 (42–67) years, *p* = 0.016) (Figure 3B). Accordingly, multiple logistic regression confirmed that age was a significant predictor of PLK-1 expression, independent of covariates such as sex, tumor grade, or treatment with biological therapy (*p* = 0.029).

**Figure 4:**
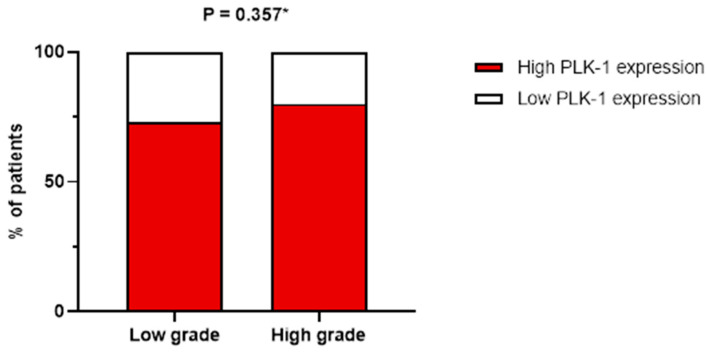
Association between tumor grade and PLK-1 expression; * chi-squared test.

**Figure 5:**
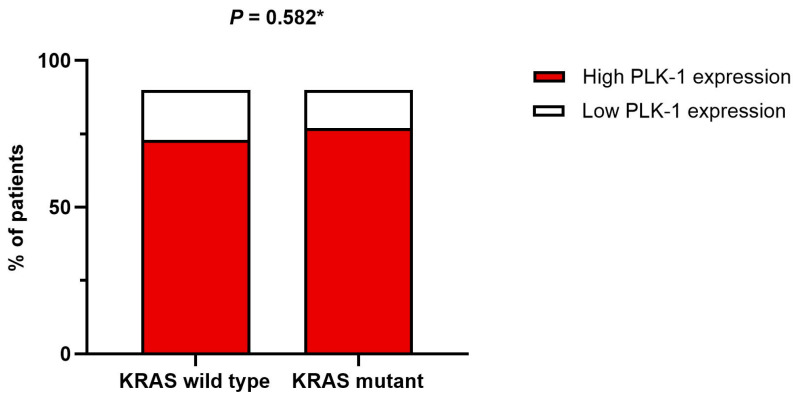
Association between KRAS mutation status and PLK-1 expression; * chi-squared test.

On the other hand, no association was found between tumor grade and PLK-1 expression. Specifically, in patients with high-grade tumors, 17 (50%) had low PLK-1 expression, whereas 66 (58.9%) had high PLK-1 expression (*p* = 0.357) (Figure 4). To further delineate differences in PLK-1 expression among CRC patients, we compared PLK-1 expression depending on KRAS mutation status. Patients with mutant KRAS and those with wild-type KRAS did not differ with respect to PLK-1 expression (55 (73.3%) vs. 48 (77.4%), *p* = 0.582) (Figure 5). Similarly, no difference in PLK-1 expression was found between left-sided and right-sided CRC (82 (73.2%) vs. 29 (87.9%), *p* = 0.081).

**Figure 6:**
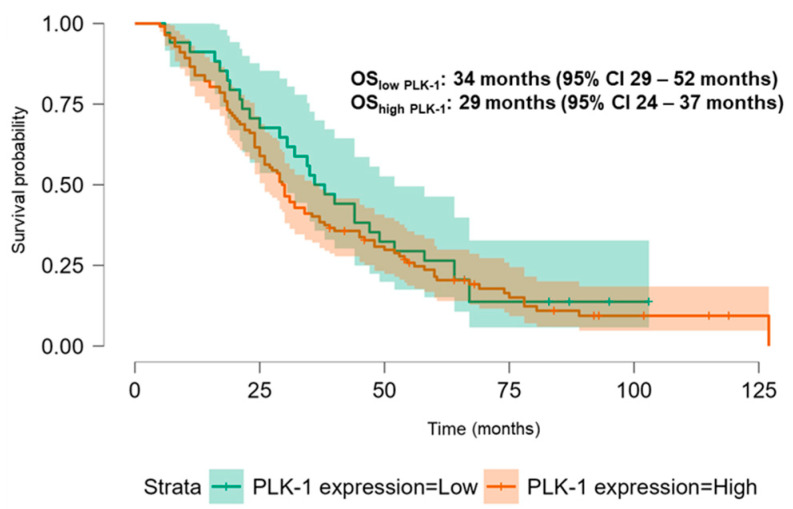
Kaplan–Meier curve showing overall survival differences based on PLK-1 expression.

**Figure 7:**
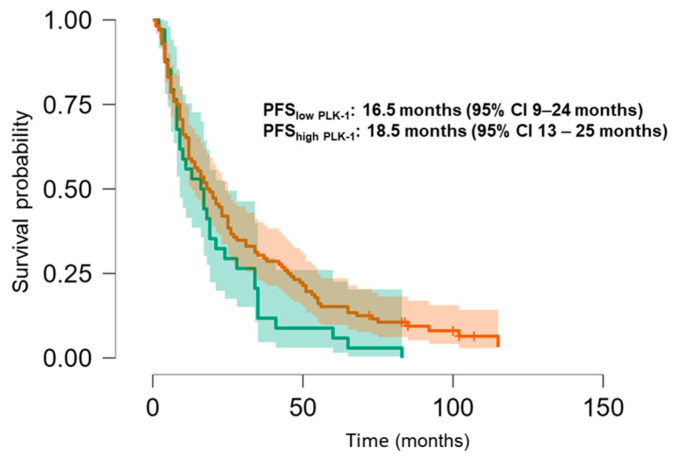
Kaplan–Meier curve showing progression-free survival differences based on PLK-1 expression.

**Figure 8:**
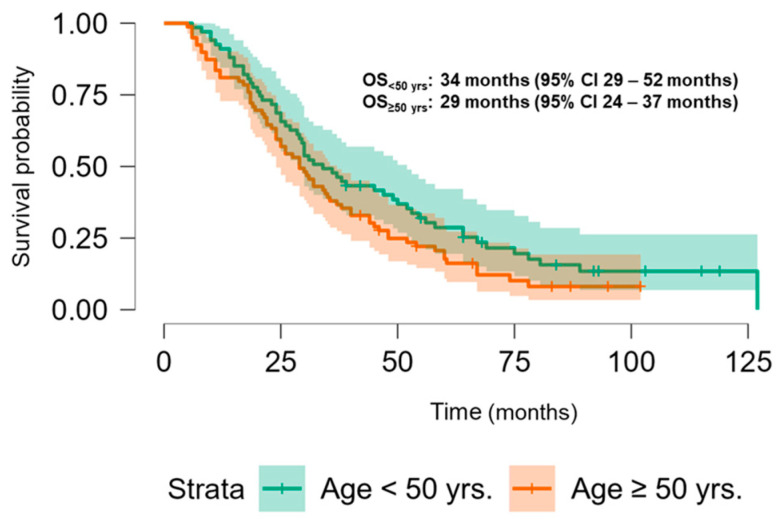
Kaplan–Meier curve showing overall survival differences based on age group.

**Figure 9:**
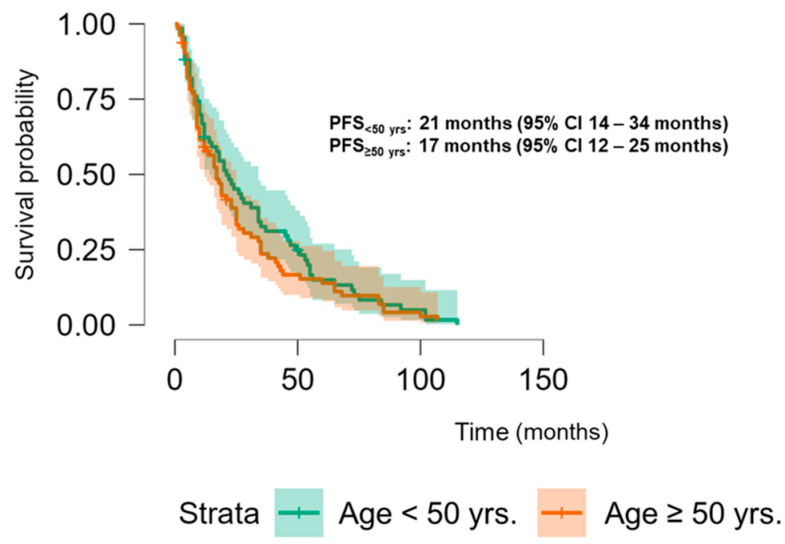
Kaplan–Meier curve showing progression-free survival differences based on age group.

The Kaplan–Meier analysis revealed no significant association between PLK-1 expression and OS (37 months (95% CI 30.5–52 months) vs. 29.8 months (95% CI 26–37 months), *p* = 0.448; see Figure 6) or PFS (16.5 months (95% CI 9–24 months) vs. 18.5 months (95% CI 13–25 months), *p* = 0.397; see Figure 7). To further investigate the potential predictive value of PLK-1 expression in terms of OS/PFS, while controlling for additional variables such as age, sex, ECOG status, and the presence of metastasis at diagnosis, we employed Cox proportional hazards regression. This analysis also indicated no significant association between PLK-1 expression and OS (*p* = 0.598) or PFS (*p* = 0.611). However, the analysis showed that poor ECOG status at diagnosis was associated with worse OS, with a hazard ratio (HR) of 4.37 (95% CI: 1.35–14.09, *p* = 0.014). Finally, Kaplan–Meier analysis showed no difference in OS (34 months (95% CI 29–52 months) vs. 29 months (95% CI 24–37 months), *p* = 0.104) (Figure 8) or PFS (21 months (95% CI 14–34 months) vs. 17 months (95% CI 12–25 months), *p* = 0.423) (Figure 9) between the designated age groups. Furthermore, the Cox proportional hazards regression model, which compared EOCRC and LOCRC with respect to PFS and OS whilst adjusting for tumor location, tumor grade, and the presence of liver metastases, showed that right-sided tumors were associated with worse OS independent of the above-noted covariates (HR 2.05, 95% CI 1.31–3.20, *p* = 0.002), while there was no statistically significant association regarding PFS (*p* = 0.118). Finally, when we performed Kaplan–Meier analysis on a subset of patients younger than 50 years, no difference in OS (58 months (95% CI 17–67 months) vs. 34 months (95% CI 27–53 months), *p* = 0.473) or PFS (11 months (95% CI 6–34 months) vs. 21 months (95% CI 14–34 months), *p* = 0.136) was found depending on PLK-1 expression.

## 4. Discussion

Although a number of studies have previously examined the prognostic role of PLK-1 in CRC, to the best of our knowledge, this is the first report of a direct comparison of PLK-1 expression between EOCRC and LOCRC with emphasis on prognostic value independent of the possible confounders. Our results indicate that age is a significant predictor of PLK-1 expression independent of sex, tumor grade, or treatment with biological therapy, but that PLK-1 is not associated with outcomes, even when covariates are taken into account. In addition, PLK-1 expression does not seem to vary significantly with tumor grade or location (left vs. right).

The search for novel prognostic factors and the development of improved therapeutic strategies in early-onset colorectal cancer (EOCRC) are critical due to its rising incidence and aggressive nature, often resulting in suboptimal clinical outcomes. Younger patients, typically in better overall health, frequently undergo more chemotherapy cycles and aggressive treatments; however, the impact on treatment outcomes remains uncertain [11,13,16]. Several lines of evidence highlight the potential relevance of PLK-1 in this context. For instance, it has been demonstrated that the expression of PLK-1 in cancer tissue is substantially higher than in normal colorectal tissue [25]. Han et al. showed that elevated expression of PLK-1 in colorectal cancer tissue is present in approximately 70% of cases, while in surrounding healthy tissue its expression was observed in less than 4%. Notably, the same authors established a correlation with disease stage, including tumor size, depth of invasion, and lymph node involvement [26].

A variety of pathophysiological mechanisms could underlie the association between PLK-1 and cancer progression. For instance, PLK-1 phosphorylates cdc25 and cyclin B1, leading to mitotic initiation [27]. Additionally, PLK-1 has been shown to promote the dissociation of cohesins and activate the anaphase-promoting complex. It also appears to play a role in the dynamic regulation of microtubules and the DNA damage response mechanism by regulating the G2-M checkpoint [28,29,30,31]. Accordingly, inhibiting PLK-1 expression has been observed to significantly reduce cell proliferation and induce apoptosis in various cancer cells. Overall, PLK-1’s involvement in mitotic regulation suggests that its dysregulation may contribute to CRC’s development and progression, although the precise mechanisms by which PLK-1 influences CRC invasion remain to be clarified. Moreover, an RNA interference screening demonstrated a synthetically lethal interaction between PLK-1 inhibition and KRAS-mutated tumors, further revealing that KRAS-mutated cell lines were more sensitive to onvansertib-mediated PLK-1 inhibition than wild-type lines [32,33]. With these findings in mind, we aimed to investigate whether PLK-1 expression is influenced by KRAS mutation status. However, no such association was identified in the present study.

The work of Rodel and colleagues is among the first to highlight the role of PLK-1 as a potential predictive factor, indicating that increased expression is associated with poorer responses of rectal cancer to radiotherapy. In patients treated with neoadjuvant chemoradiotherapy (preoperative therapy), high expression of PLK-1 was linked to poorer tumor regression and a greater likelihood of local disease recurrence. These results suggest that tumor cells with elevated PLK-1 expression present a more radioresistant phenotype [24]. Accordingly, Takahashi et al. demonstrated that PLK-1 may play a role in disease progression in advanced stages. However, no association with sex, age, histological differentiation, tumor location, or distant metastases was found. The study’s potential limitations were the small number of included patients, just 78, and the cut-off value between age groups being set at 63 years [34]. Furthermore, a crucial piece of evidence in terms of PLK-1’s role in CRC’s prognosis is a meta-analysis by Ran et al. that included 11 studies investigating the clinicopathological and prognostic impact of overexpression of PLK-1 in CRC [25]. The analysis showed that overexpression of PLK-1 was associated with poorer OS. In addition, positive PLK-1 expression was associated with lymph node metastasis, invasion, and advanced TNM stage, implying that PLK-1 expression could be an important factor in assessing the biological behavior and prognosis of colorectal cancer. However, in contrast to our results, this meta-analysis showed no association between age and PLK-1 expression. As no such association was found in this study, there is a possibility that some other signaling pathways can modulate the effect of PLK-1 expression in CRC cells in young people. In addition, perhaps a more aggressive approach to treatment in young patients compensates for a more aggressive tumor biology compared to the elderly patients, resulting in the absence of a difference in OS and PFS. This is also supported by the fact that no difference in outcomes was found between EOCRC and LOCRC in the studied population. PLK-1 has also proven promising in other cancers, such as ovarian cancer, where a correlation between PLK-1 expression and clinical stage and grade has been established [35], and in melanoma, where an association with distant metastases has been demonstrated [36].

Interestingly, Yu et al. discovered the significance of the PLK-1-MYC-CDC7 axis in chemoresistance. Resistance to oxaliplatin presents a significant challenge in the treatment of CRC and often leads to disease recurrence [37]. The aforementioned study demonstrated that the inhibition of PLK-1 significantly sensitizes cancer cells to oxaliplatin therapy, and that combined treatment with chemotherapy and PLK-1 inhibitors exhibits a synergistic effect both in vitro and in vivo, potentially opening new horizons in overcoming chemoresistance. Finally, it is worth noting that several PLK-1 inhibitors, such as BI2536, BI6727 (volasertib), and NMS-1286937 (onvansertib), have been studied in preclinical and clinical research [38,39,40,41,42]. The primary challenge was therapy resistance. Some studies did not show a benefit in overall survival, but in combined therapeutic protocols with chemotherapy, immunotherapy, or targeted therapy, they could potentially be more effective.

The inferences stemming from this research are limited by several factors. First, this study is retrospective in nature. Second, the number of patients is limited, and all patients were of Caucasian descent from two centers in Croatia, thus limiting the results to that population. Finally, a longer follow-up could give us better insight into the associations between PLK-1 expression and outcomes.

## 5. Conclusions

In conclusion, this analysis demonstrated higher PLK-1 expression in patients younger than 50 years and found that age is a significant predictor of PLK-1 expression independent of covariates. Consistent with previous research, our investigation showed that patients younger than 50 years have a higher prevalence of high-grade carcinomas; however, a statistically significant association between PLK-1 expression and tumor grade was not established. Regarding the main objective, no statistically significant association was found between PLK-1 expression and PFS or OS. Given the increasing incidence of EORTC and unsatisfactory treatment outcomes, the discovery of new prognostic and predictive factors could help in developing new combined treatment strategies. Despite the previous research and promising data considering PLK-1 a prognostic/predictive factor, it seems that it has no clear clinical effect in the setting of mCRC. Although several molecular mechanisms suggest that PLK-1 could have possible prognostic and predictive value in mCRC patients, the results of our study did not confirm this, since we did not notice any effect of PLK-1 on survival or progression.

PLK-1 could certainly be a targeted therapy. Nonetheless, the importance of PLK-1 should be investigated in further research in order to obtain more comprehensive knowledge regarding its possible predictive and prognostic effects in mCRC patients. Specifically, the significance of PLK-1 in relation to outcomes may significantly vary among patients depending on the stage of the disease as well as their origin and other relevant characteristics. It is also important to establish whether PLK-1 is interrelated with some of the established biomarkers related to CRC.

## Figures and Tables

**Table 1:** PLK-1 staining intensity, percentage of positive tumor cells, and weighted score in histopathological samples of metastatic colorectal cancer.

	Staining Intensity				% Positive Tumor Cells				Weighted Score		
	Total N (%)	<50 N (%)	≥50 N (%)		Total N (%)	<50 N (%)	≥50 N (%)		Total N (%)	<50 N (%)	≥50 N (%)
**No reaction (0)**	2 (1.4)	1 (1.5)	1 (1.3)	**0–5% (0)**	2 (1.4)	1 (1.5)	1 (1.3)	**0–3**	16 (11.0)	8 (11.9)	8 (10.1)
**Weak (1)**	16 (11.0)	8 (11.9)	8 (10.1)	**6–25% (1)**	8 (5.5)	1(1.5)	7 (8.9)	**4–6**	60 (41.1)	16 (23.9)	44 (55.7)
**Moderate (2)**	64 (43.8)	19 (28.4)	45 (57.0)	**26–50% (2)**	22 (15.1)	5 (7.5)	18 (22.8)	**7–9**	18 (12.3)	8 (11.9)	10 (12.7)
**Intense (3)**	64 (43.8)	39 (58.2)	25 (31.6)	**51–75% (3)**	59 (40.4)	22 (32.8)	36 (45.6)	**12**	52 (35.6)	35 (52.2)	17 (21.5)
				**>75% (4)**	55 (37.7)	38 (56.7)	17 (21.5)				

**Table 2:** Baseline characteristics of the studied population.

Parameter	Total Population (N = 146)	Age < 50 years (N = 67)	Age ≥ 50 years (N = 79)	*p* *
Age, years	56 (44–68)	43 (40–47)	67 (62–75)	0.839
Female sex, n (%)	73 (50)	44 (65.7)	29 (36.7)	<0.001
Follow-up, months	38 (23–67)	39 (24–73)	37 (22–60)	0.459
ECOG at the time of diagnosis, n (%)				
0	60 (40.8)	26 (76.5)	34 (48.6)	
1	40 (27.2)	8 (23.5)	32 (45.7)	0.019
2	4 (2.7)	0 (0)	4 (5.7)	
Biological therapy, n (%)				
No therapy	33 (22.6)	9 (14.1)	24 (32)	0.560
Bevacizumab	68 (48.9)	35 (54.7)	33 (44.0)	0.273
EGFR inhibitor	38 (27.3)	20 (31.3)	18 (24.0)	0.435
Primary tumor localization, n (%)				
Left	113 (77.4)	53 (79.1)	59 (74.7)	0.273
Right	34 (23.3)	14 (20.9)	20 (25.3)	
Metastatic at diagnosis, n (%)	67 (45.9)	23 (34.3)	43 (54.4)	0.015
Metastasis localization, n (%)				
Liver	106 (72.6)	57 (85.1)	49 (62.0)	0.002
Lungs	76 (52.1)	39 (58.2)	37 (46.8)	0.170
Bones	20 (13.7)	11 (16.4)	9 (11.4)	0.379
Peritoneum	34 (23.3)	21 (31.3)	13 (16.5)	0.034
Grade, n (%)				
Low	64 (43.8)	12 (17.9)	51 (64.6)	<0.001
High	82 (56.2)	55 (82.1)	28 (35.4)	
RAS mutation, n (%)	75 (51.4)	35 (56.5)	40 (53.3)	0.715
BRAF mutation, n (%)	4 (2.7)	0 (0)	4 (5.1)	0.140

* Chi-squared test or Mann–Whitney test, as appropriate. Data presented as the median (interquartile range) or n (%). Abbreviations: BRAF: v-Raf Murine Sarcoma Viral Oncogene Homolog B1; ECOG: Eastern Cooperative Oncology Group; EGFR: Epidermal Growth Factor Receptor; RAS: Rat Sarcoma Virus.

## Data Availability

The original contributions presented in the study are included in the article, further inquiries can be directed to the corresponding author.
